# Quantitative birefringence microscopy for imaging the structural integrity of CNS myelin following circumscribed cortical injury in the rhesus monkey

**DOI:** 10.1117/1.NPh.8.1.015010

**Published:** 2021-03-22

**Authors:** Nathan Blanke, Veronica Go, Douglas L. Rosene, Irving J. Bigio

**Affiliations:** a Boston University, Neurophotonics Center, Department of Biomedical Engineering, Boston, Massachusetts, United States; b Boston University School of Medicine, Department of Anatomy and Neurobiology, Boston, Massachusetts, United States; c Boston University, Department of Electrical and Computer Engineering, Boston, Massachusetts, United States

**Keywords:** birefringence, microscopy, myelin, structure, polarized light, stroke, Wallerian degeneration

## Abstract

**Significance:** Myelin breakdown is likely a key factor in the loss of cognitive and motor function associated with many neurodegenerative diseases.

**Aim:** New methods for imaging myelin structure are needed to characterize and quantify the degradation of myelin in standard whole-brain sections of nonhuman primates and in human brain.

**Approach:** Quantitative birefringence microscopy (qBRM) is a label-free technique for rapid histopathological assessment of myelin structural breakdown following cortical injury in rhesus monkeys.

**Results:** We validate birefringence microscopy for structural imaging of myelin in rhesus monkey brain sections, and we demonstrate the power of qBRM by characterizing the breakdown of myelin following cortical injury, as a model of stroke, in the motor cortex.

**Conclusions:** Birefringence microscopy is a valuable tool for histopathology of myelin and for quantitative assessment of myelin structure. Compared to conventional methods, this label-free technique is sensitive to subtle changes in myelin structure, is fast, and enables more quantitative assessment, without the variability inherent in labeling procedures such as immunohistochemistry.

## 1 Introduction

The myelin sheath that wraps and insulates axons of the central nervous system (CNS), as well as peripheral nerves, is essential for the proper function of mammalian neural signaling. Once formed, myelin is a compact, multilayered assembly of tightly wrapped lipid membranes held together by protein molecules. During the myelination process in the CNS, oligodendrocytes, the glial cells responsible for producing myelin, send out processes that wrap around axons as many as 160 times,^1^ with the number of lamellae being approximately linearly related to the axon diameter.^2^ As the multilayer myelin sheath compacts, oligodendrocyte cytoplasm is displaced to the periphery of the processes, resulting in a tight apposition of concentric lipid bilayers of glial-cell membrane around the axon. This unique structure facilitates efficient neuronal communication through the well-established phenomenon of saltatory conduction,^3^ and its structural anisotropy is the basis for contrast in the optical imaging reported here.

In demyelinating diseases of the CNS, damage to myelin, or the oligodendrocytes that produce it, is primary to the disease process and can occur as a result of autoimmune reactions, exposure to viruses or toxins, metabolic disorders, and hypoxic or ischemic challenge.^4^ The classical example of a demyelinating disease is multiple sclerosis (MS), where CNS inflammation leads to large-scale insults to myelin,^5^ causing a variable range of motor, cognitive, and neuropsychiatric symptoms depending on location.^6^ Beyond MS, increasing evidence points toward myelin breakdown as an important factor in other major neurodegenerative diseases, including age-related cognitive decline,^7^^–^^9^ Alzheimer’s disease (AD),^10^^–^^14^ and stroke.^15^^–^^17^

While the pathological features of neurodegenerative diseases are various, many are characterized by structural changes to myelin. In MS and other demyelinating diseases, myelin undergoes complete structural breakdown and forms into collections of small myelin vesicles that are eventually phagocytosed by microglia or peripheral macrophages.^18^ This vesiculated myelin debris is a hallmark of an active demyelinating lesion.^7^^,^^19^^,^^20^ Similarly, in the events that follow ischemic stroke, as axons of dying neurons lose their metabolic support and undergo Wallerian degeneration, myelin debris structures are also formed.^21^ In the CNS, large amounts of myelin debris cannot be efficiently cleared by resident microglia and often persist for many years in severely affected regions of the human brain.^22^ Although it is not known how well they represent human pathology, in animal models of aging and AD, myelin pathology is typically more subtle, with alterations to myelin consisting of splitting of myelin lamellae, ballooning of individual myelin internodes, and the formation of redundant sheaths.^8^^,^^23^^–^^25^

Of the many imaging methods available for studying myelin and its pathology in the brain, electron microscopy (EM) is the gold-standard for evaluation of myelin integrity, as it allows for unequaled resolution of normal and abnormal myelin structure of individual myelin sheaths.^18^^,^^23^^,^^26^ However, EM is not scalable for analysis of whole-brain sections, and it requires special fixation that is difficult to perform and is often incompatible with other methods. In attempts to meet the need for efficient myelin imaging across large territories in brain tissues not fixed for EM, a number of histochemical staining and immunohistochemical (IHC) labeling techniques have been developed.^27^^–^^30^ These techniques are convenient for delineation of white and gray matter regions in brain tissue and for crude demonstration of large-scale demyelination in white matter (e.g., cuprizone-diet-induced demyelination in animals^31^^,^^32^), but the protocols are often complex, suffer from inherent variability, and are not suitable for structural characterization of individual myelin sheaths. Furthermore, IHC labeling of myelin faces unique challenges, as the compact layering of lipid membrane prevents IHC probes from uniformly accessing binding sites, even with the use of harsh permeabilization agents.^33^^,^^34^ Of the existing label-based methods for myelin imaging, fluorescent lipophilic dyes, including FluoroMyelin Red,^35^ have shown the most promise. These dyes do not require membrane permeabilization, leaving myelin lipids intact, and can be imaged at high-resolution with confocal fluorescence microscopy. Despite these benefits, there is always a significant amount of fluorophore present that is not specific to myelin, resulting in background signal. In addition, confocal microscopy imposes limitations on imaging speed and field of view.

Importantly, none of the label-based approaches are suitable for quantitative imaging of myelin structure, especially in disease processes that involve more subtle myelin pathology, where detailed imaging of individual myelinated axons is required. For characterization of myelin status in neurodegenerative diseases, high-resolution, label-free methods are necessary for assessing and quantifying myelin structure and pathology. Moreover, to facilitate histopathology of entire brain sections, a label-free method should ideally be widefield (rather than point scanning) and should provide high-contrast images rapidly. Current label-free, optical methods for imaging myelin include spectral confocal reflectance (SCoRe) microscopy,^34^^,^^36^^,^^37^ third-harmonic generation microscopy,^38^^,^^39^ and coherent anti-Stokes Raman scattering (CARS) microscopy.^40^^–^^44^ While these techniques all share the potential for label-free images of myelin *in vivo* (albeit over a small field-of-view), when used for analysis of *ex vivo* tissues in a histopathological setting, the requirement of point scanning to form an image renders them slow and inefficient for myelin imaging across multiple brain regions or large brains. In addition, these imaging methods all require expensive and complex multilaser systems, limiting their applicability for general use.

To address the need for a quantitative, rapid, and practical method for high-resolution imaging of myelin structure and pathology, we have optimized quantitative birefringence microscopy (qBRM) as a label-free technique for histopathological assessment of myelin breakdown across large brain regions, with resolution down to the level of individual myelin sheaths. The earliest reports of using birefringence (or polarized-light) microscopy for imaging myelin degeneration in peripheral nerves were published decades ago.^45^^–^^47^ Those early reports, however, described only qualitative imaging. More recently, qBRM has been demonstrated,^48^^,^^49^ enabling determination of the relative retardance and local orientation of structural anisotropy for every pixel in an image. Thus far, for applications in neuroscience, qBRM has been successfully used to map the connectivity of large myelinated fiber tracts in the human brain^50^ and has been demonstrated for imaging directionality of myelinated axons in mouse and developing human brain tissue.^51^ To our knowledge, however, qBRM has not yet been implemented for detailed structural characterization of myelin degradation at single-axon resolution. Here, we demonstrate the benefits of qBRM applied to study the degradation of myelin, following circumsribed cortical damage in a nonhuman primate model of stroke.^52^

## 2 Methods

### 2.1 Imaging Birefringence of Myelin

Birefringence microscopy is a method for imaging contrast based on the birefringent structures in specimens. By illuminating the specimen with light of one polarization state while detecting the orthogonal polarization state (linear or elliptical), all light is blocked from detection, unless it has propagated through a birefringent structure that alters its polarization state. In the resulting images, label-free birefringent structures appear bright on a dark background, exhibiting benefits similar to dark-field microscopy.

Birefringence refers to the property of anisotropic media, by which light propagating in specific directions experiences different indices of refraction for different polarization states of the incident light (orientations of the optical electric field), due to the inherent difference in polarizability of the medium along different axes. For uniaxial birefringent media, the optic axis is defined as the axis along which propagating light will experience no birefringence.^53^^,^^54^ Conversely, when the direction of light propagation is perpendicular to the optic axis, the light will experience the maximum variation in refractive index for optical electric-field orientations parallel and orthogonal to the optic axis. These structural axes are termed the “ordinary” and “extraordinary” axes. That is, light polarized parallel to the optic axis will experience the extraordinary optical index, $n_{e}$, whereas the orthogonal polarization will experience the ordinary index, $n_{o}$. As a result, a relative phase delay, or optical retardance, accumulates between the two polarization components, with the magnitude of the retardance being proportional to the product of the refractive-index difference, $\Delta n=|n_{e}-n_{o}|$, and the thickness of the birefringent specimen, L. This retardance leads to a wavelength-dependent change in the ellipticity of the light polarization-state upon exiting the sample, which, when normalized to wavelength, λ, yields the relative retardance in waves (or fraction of a wave):

$$\delta =\frac{\Delta n\cdot L}{\lambda }.$$ (1)

Using cross-polarized optics (see Sec. 2.2.1), an arbitrary change in polarization state can be viewed or imaged on a camera to provide label-free images of sample birefringence.

**Fig. 1:**
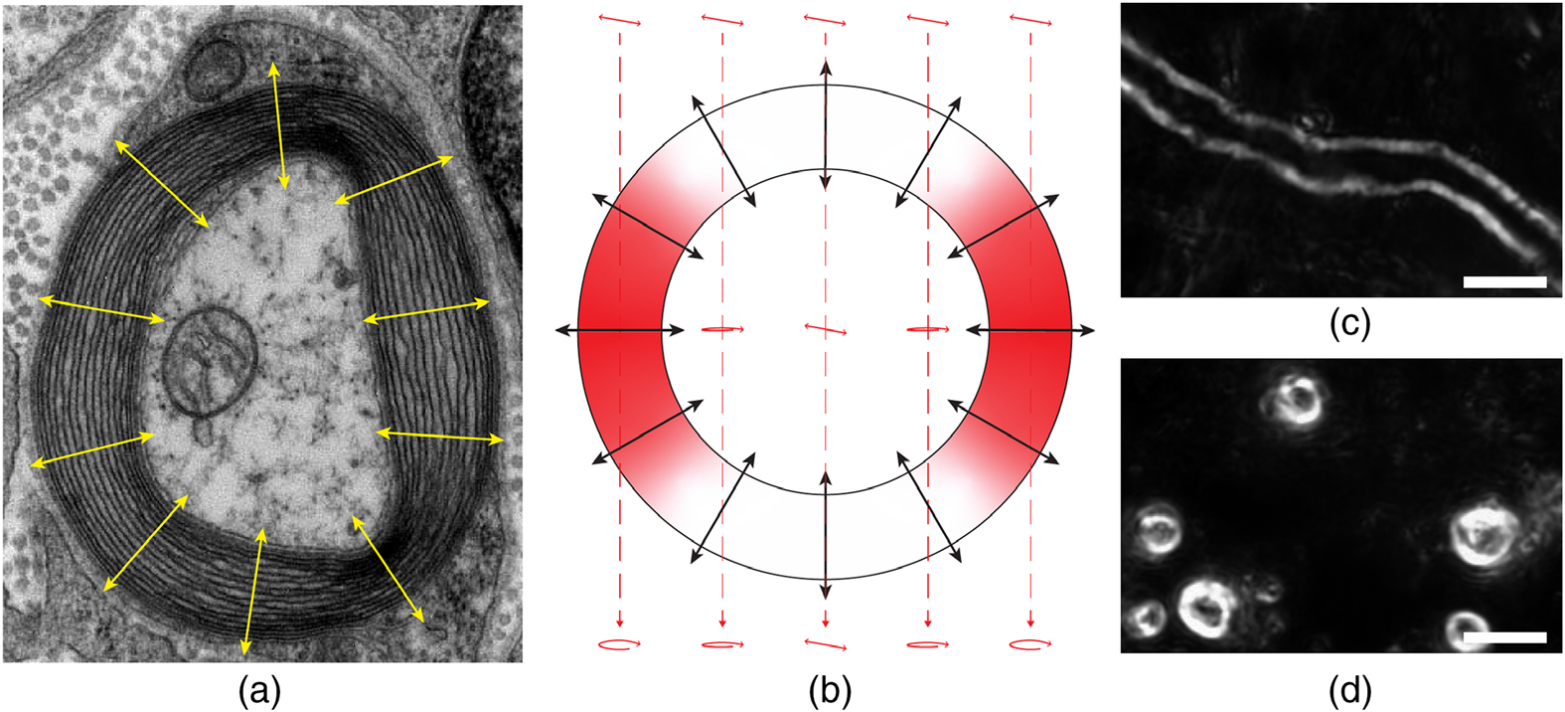
Optic axis of myelin. (a) Transmission electron micrograph of a myelinated axon in cross-section. The myelin optic axis is indicated with yellow arrows. Image by Roadnottaken, modified,^56^ (CC BY-SA 3.0). (b) Cross-sectional diagram for downward propagation of linearly polarized light through a myelin sheath that is longitudinally oriented within the tissue section. Areas of image contrast are indicated with red shading. (c) Longitudinally and (d) transversely oriented myelinated axons from cortical gray matter of a young and healthy rhesus monkey imaged with birefringence microscopy (60× oil objective, NA 1.35). Scale bars: 10  μm.

Due to its highly anisotropic structure, myelin exhibits strong birefringence.^55^ The myelin sheath, with its multiple, concentric layers of oligodendroglial lipid membrane [Fig. 1(a)], exhibits cylindrical symmetry. Consistent with the radially aligned lipid molecules that comprise the sheath, the myelin optic axis is radial with respect to the longitudinal axis of the axon [yellow arrows in Fig. 1(a) and black arrows in Fig. 1(b)].^57^^,^^58^ Therefore, for a myelinated axon that is oriented longitudinally within a tissue section [Fig. 1(c)], birefringence contrast is generated along the sides of the sheath, where the myelin optic axis is transverse to the direction of light propagation [represented by the red areas of the orthogonal view depicted in Fig. 1(b)]. For the top and bottom portions of the same myelin sheath, where the myelin optic axis is parallel to the direction of light propagation, polarized light passes through unaltered. On the other hand, for a myelinated axon oriented transversely within a tissue section [Fig. 1(d)], contrast is exhibited for the full circular cross-section since the myelin optic axis for these axons is always transverse to the direction of light propagation. When viewing longitudinal axons, the enhanced contrast of the sides of the myelin sheath is beneficial for structural imaging, as the segment of the myelin sheath exhibiting maximum image contrast resides at one focal plane and can therefore be brought into sharp focus. This imaging feature aids in the identification of structural abnormalities along the myelin profile and helps to facilitate the acquisition of high-resolution, volumetric imaging data.

### 2.2 Instrumentation

Our birefringence microscope is a modified adaptation of a standard brightfield microscope (Olympus IX-81). It houses a full set of strain-free air objectives (ranging from 4× to 40×), as well as a 60× oil-immersion objective, to provide flexibility in the field-of-view and optical resolution used for imaging. A motorized XY-scanning stage (Thorlabs MLS203-1) and motorized objective Z-positioning facilitate automated and repeatable acquisition of volumetric imaging data. For polarization control, the microscope is equipped with custom-mount polarization optics in both the illumination and detection arms. The pairing of a linear polarizer (Thorlabs LPVISE100-A) on a motorized rotational stage (Thorlabs K10CR1), stacked with a stationary quarter-wave plate (Thorlabs WPQ10M-633), results in independent variable-ellipticity polarization control on both the illumination (polarizer) and detection (analyzer) sides. This system enables rapid implementation of two complementary configurations for birefringence microscopy of myelin. Section 2.2.1 addresses real-time imaging of the sample between cross-circular-polarized optics, and Sec. 2.2.2 details quantitative birefringence imaging with multiple images.

#### 2.2.1 Cross-circular-polarized birefringence microscopy

**Fig. 2:**
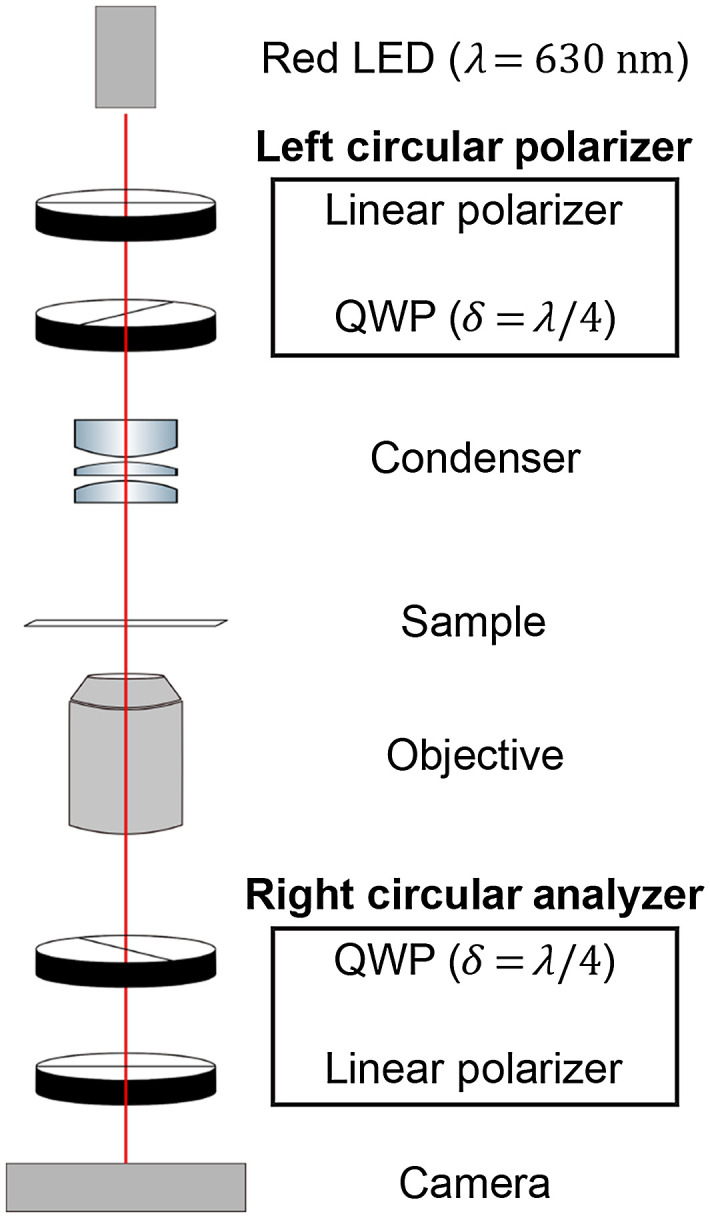
CCP-BRM for rapid qualitative imaging. The resulting images of sample birefringence are independent of specimen orientation and can be viewed directly with a camera or through the microscope eyepieces. (QWP, quarter-wave plate).

Rapid histopathological assessment of myelin is carried out with a circular polarizer and a circular analyzer (Fig. 2), of opposite handedness: “cross-circular-polarized birefringence microscopy” (CCP-BRM). With the use of circular polarizer and analyzer, as opposed to simply crossed-linear polarizers, orientation-independent image contrast is generated in real-time. This method was first demonstrated as early as 1966 for orientation-independent imaging of myelin birefringence in the nervous system,^47^ yet its robustness for label-free histopathology of myelin in brain tissue has not been fully realized. Compared to other groups that have used liquid-crystal variable retarders for maximizing speed during quantitative birefringence imaging,^59^^,^^60^ our system has been designed with zero-order quarter-wave plates to achieve better extinction ratios and to maximize contrast for the smallest birefringent structures (i.e., the smallest myelinated axons) that can be imaged with a diffraction-limited optical system. This CCP-BRM imaging modality provides a unique method for efficiently imaging birefringence contrast of myelin, without requiring the series of polarization images and image postprocessing steps that are needed to generate comparable images with qBRM (see Sec. 2.2.2).

#### 2.2.2 Quantitative birefringence microscopy

**Fig. 3:**
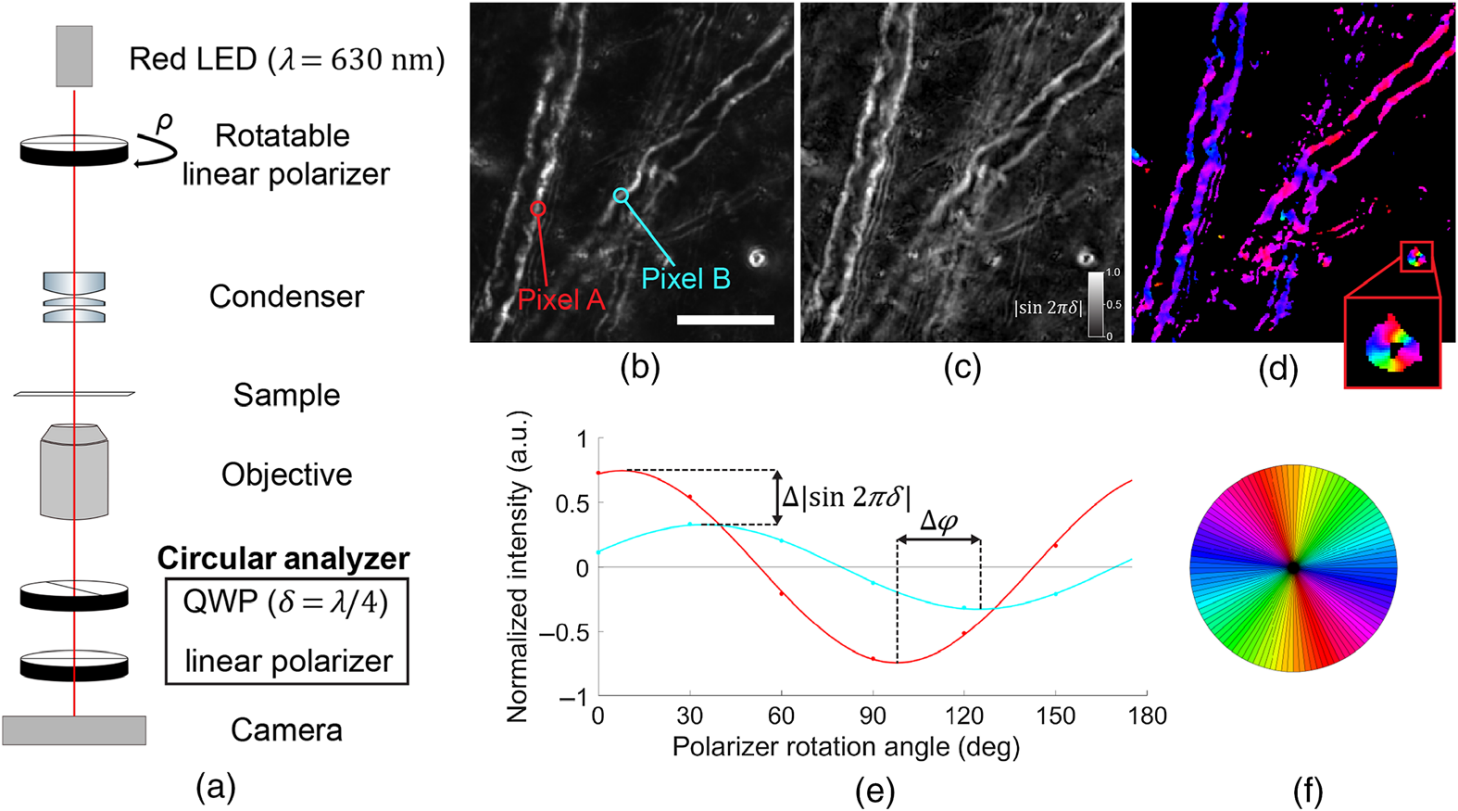
qBRM. (a) Setup for qBRM. Images are taken during stepwise rotation of the illumination-side linear polarizer. (QWP, quarter-wave plate) (b) CCP-BRM image of normal myelin in rhesus monkey gray matter, taken with the setup shown in Fig. 2 (40× objective, NA 0.75). Two large myelinated axons are seen at slightly different in-plane orientations, with example pixels along their edges highlighted. Scale bar: 20  μm. (c) Retardance (|sin(2πδ)|) map (inset: grayscale color bar) and (d) in-plane optic-axis orientation (φ) map (inset: transversely sectioned myelin sheath) generated with qBRM. (e) Quantitative pixel traces (DC-subtracted and normalized), obtained by rotation of the linear polarizer and after correction, for the two individual pixels identified by color in (b). Images were taken at 30-deg increments before fitting. (f) Color scale used for display of optic-axis orientation in (d).

qBRM is a method for extraction of birefringence parameter maps from anisotropic samples by imaging with monochromatic light at a sequence of polarization states.^49^^,^^61^ When studying disease processes in anisotropic tissues, these birefringence parameters provide meaningful insights into the changes in molecular order and organization that accompany structural degradation, as will be illustrated below for myelin. For qBRM, we employ one of the simplest configurations, utilizing a rotating linear polarizer for illumination and a circular-polarization analyzer for detection^48^ [Fig. 3(a)]. In this framework, an image sequence is taken during stepwise rotation of the illumination polarizer, and the birefringence intensity of each pixel varies sinusoidally, according to

$$I=\frac{I_{0}}{2}[1+\sin(2\rho -2\varphi )\sin(2\pi \delta )],$$ (2)

where $I_{0}$ is the intensity incident on the sample, ρ is the rotation angle of the linear polarizer,   φ is the angular orientation of the myelin optic axis in the imaging plane, and δ is the relative retardance given in Eq. (1). When the rotating linear polarizer is at 45 deg to the myelin optic axis, in either direction (ρ=φ±45  deg), the signal is at its maximum and minimum, $I=\frac{I_{0}}{2}\pm \frac{I_{0}}{2}\;\sin(2\pi \delta )$. When the rotating linear polarizer is either parallel with (ρ=φ) or orthogonal to (ρ=φ+90  deg) the myelin optic axis, within the polarization plane, only the circular analyzer contributes to the detected intensity, and the signal is at its DC level, $I=\frac{I_{0}}{2}$. With images taken at multiple, discrete polarizer rotation angles, nonlinear least-squares regression can be used to fit the resulting intensity trace and recover the amplitude and phase for every pixel, providing birefringence parameter maps of retardance, |sin(2πδ)|, and optic-axis orientation, φ, respectively. The retardance map provides a measure of local density (and degree of alignment) of myelin, whereas the optic-axis orientation map provides a measure of the two-dimensional (2D) in-plane orientation.

In our typical workflow, the sample is first imaged with CCP-BRM to identify regions of interest and to bring meaningful structures into focus for quantitative analysis. For the subsequent analysis with qBRM, one image set is taken of the sample during rotation of the linear polarizer [Fig. 3(a)], and a second image set, used for correction, is taken of an empty region in the sample plane. The linear polarizer is rotated in 30-deg steps over a 180-deg range, with 10-image bursts (for averaging during correction) taken at each of the six angular positions. To correct for variations in image intensity, due to system or background noise, qBRM image sets are both flat-field and background corrected. Flat-field correction helps to remove artifacts caused by uneven illumination and dust particles along the optical path, whereas background correction removes the component of the measured signal, during polarizer rotation, that is not due to tissue birefringence. This background component is largely a consequence of using a wavelength-specific quarter-wave plate with a red LED source of significant bandwidth, causing the circular analyzer to introduce a small degree of ellipticity into the beam. This is not a concern during CCP-BRM, as any ellipticity introduced by the polarizer is canceled by the analyzer. For qBRM, however, this effect causes different orientations of the optical polarization to pass the analyzer with varying efficiency. In practice, this background component is typically about 1/10 to 1/5 of the dynamic range of the signal measured from an individual myelin sheath and is subtracted from Eq. (2) to yield the DC-subtracted pixel trace, $I=\frac{I_{0}}{2}[\sin(2\rho -2\varphi )\sin(2\pi \delta )]$. For the final correction step, the amplitude of the birefringence signal, $\frac{I_{0}}{2}|\sin(2\pi \delta )|$, for each pixel, is normalized to the maximum value across the image. This removes the dependence on the illumination intensity, $\frac{I_{0}}{2}$, allowing the amplitude to be analyzed directly as a measure of retardance, |sin(2πδ)|. For other sources of noise, such as finite polarizer extinction-ratios and reflections at air–glass interfaces, we have observed only a modest level of background that does not impact the ability to image strongly birefringent myelin. After correction steps, the final sinusoidal pixel traces are analyzed for extraction of the birefringence parameters on a pixel-by-pixel basis.

In brain tissue, high-resolution qBRM enables detailed characterization of both structurally normal [Figs. 3(b)–3(d)] and abnormal (Fig. 5) myelin. Figure 3(b) shows a CCP-BRM image of normal myelin, taken within cortical gray matter from a young and healthy monkey. Two pixels that correspond to the edges of two different myelin sheaths are indicated for illustration of the quantitative method. The qBRM parameter maps of retardance (|sin(2πδ)|) [Fig. 3(c)] and optic-axis orientation (φ) [Fig. 3(d)] are generated by extracting the amplitude and phase from the quantitative pixel traces [examples in Fig. 3(e)] that are recorded by rotation of the linear polarizer. In Fig. 3(e), the difference in density and optic-axis orientation, for the two pixels indicated in Fig. 3(b), is shown as a difference in sinusoidal amplitude (Δ|sin(2πδ)|) and phase (Δφ), respectively. The retardance map [Fig. 3(c)] was normalized by the maximum retardance value in the image and is displayed with a grayscale color map. For the optic-axis orientation map [Fig. 3(d)], pixels are assigned colors based on the color wheel shown in Fig. 3(f), with black being assigned to pixels that did not meet the normalized retardance threshold of 0.25 (i.e., pixels that contain out-of-focus or weakly birefringent structures). Note the transversely sectioned myelin sheath in the inset of Fig. 3(d), which closely mirrors the color wheel shown in Fig. 3(f).

### 2.3 Sample Tissue Preparation

#### 2.3.1 Animal model

The brain tissue used in this study comes from a nonhuman primate (rhesus monkey) model for evaluating impairment and recovery of function following circumscribed cortical damage similar to stroke. In these animals, a lesion was induced in the region of the motor cortex responsible for fine-motor control of the dominant hand, and the recovery of the animal’s grasp patterns was monitored as a function of age.^52^ Following completion of behavioral testing and euthanasia, the monkey brain was perfusion-fixed, harvested, cryoprotected, flash frozen, and cut into an interrupted series of 30-μm thick frozen sections. These were placed in 0.1M phosphate buffer with 15% glycerol and stored at −80°C until removal from long-term storage for imaging. This method has been shown to provide long-term preservation of tissue architecture and histochemical reactivity.^62^^,^^63^

#### 2.3.2 Sample preparation for birefringence microscopy of myelin

For myelin imaging with birefringence microscopy, monkey brain sections were removed from cryogenic storage, rinsed with phosphate-buffered saline (PBS), and mounted on microscope slides with a mounting medium of 70% glycerol (30% PBS) to approximately match the refractive index of lipids. Lipids constitute the primary source of scattering in brain tissue, and index-matching minimizes the scattering background while preserving birefringence contrast for myelin. It is important to note that other popular techniques for reducing scattering in brain tissue, which are based on extraction of lipids (e.g., CLARITY^64^), are unsuitable for structural imaging of myelin.

To cross-validate the specificity of birefringence microscopy for imaging myelin, we stained a few of the brain sections with FluoroMyelin™ Red (ThermoFisher Scientific). Free-floating sections were placed into a 1:200 dilution of FluoroMyelin in PBS for 20 min. They were then rinsed three times for 10 min each in PBS before being mounted on microscope slides with 70% glycerol.

## 3 Results

### 3.1 Birefringence Microscopy is Highly Selective to Myelin Structure in Brain Tissue

**Fig. 4:**
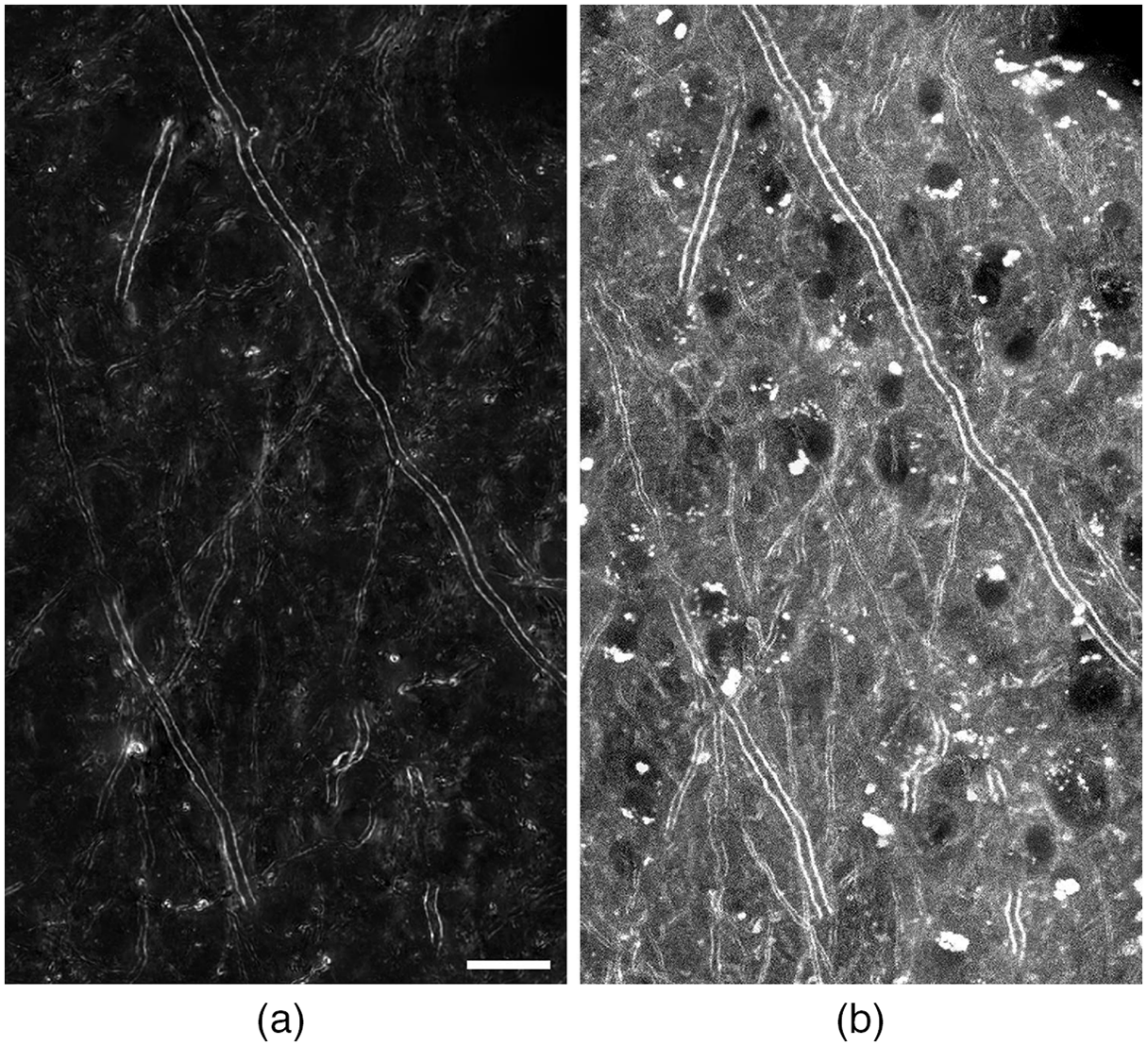
Validation of birefringence microscopy with fluoromyelin red. Rhesus monkey brain tissue was stained with fluoromyelin red, and myelinated axons within cortical gray matter were then imaged with (a) label-free CCP-BRM (40× objective, NA 0.75) and (b) confocal fluorescence microscopy (20× objective, NA 0.75). Scale bar: 20  μm. Even with the benefit of confocal microscopy, significant nonspecific fluorescence can be seen as broad background and bright spots of unbound stain in (b).

To validate birefringence microscopy, monkey brain sections stained with FluoroMyelin Red were imaged with both CCP-BRM [Fig. 4(a)] and confocal fluorescence microscopy [Fig. 4(b)] in a region of cortical gray matter. FluoroMyelin is a lipophilic dye that has a strong affinity for lipid-rich myelin, and although this stain exhibits limited diffusivity in the heavily myelinated monkey brain [bright clusters of stain seen throughout Fig. 4(b)], it remains the best conventional method for high resolution, specific imaging of myelin. Since myelinated axons can have various orientations with respect to the sectioning plane, and thus traverse different depths within any general region of the brain, to image myelin at high resolution often requires acquisition of volumetric images (z-stacks). To generate the resulting 2D projections of the birefringence and FluoroMyelin z-stacks, shown in Fig. 4, each z-stack (covering the 30-μm thickness of the sections in 2-μm increments) was processed with focus-stacking software (Adobe Photoshop), providing a single, in-focus image across the entire field of view. With these images, we confirmed that label-free birefringence microscopy is selective to myelin in the brain, and that for longitudinal (or approximately longitudinal) axons, birefringence contrast is generated along the sides of the myelin sheath, as discussed in Sec. 2.1.

### 3.2 Birefringence Microscopy Reveals Presence of Myelin Debris Following Cortical Injury

**Fig. 5:**
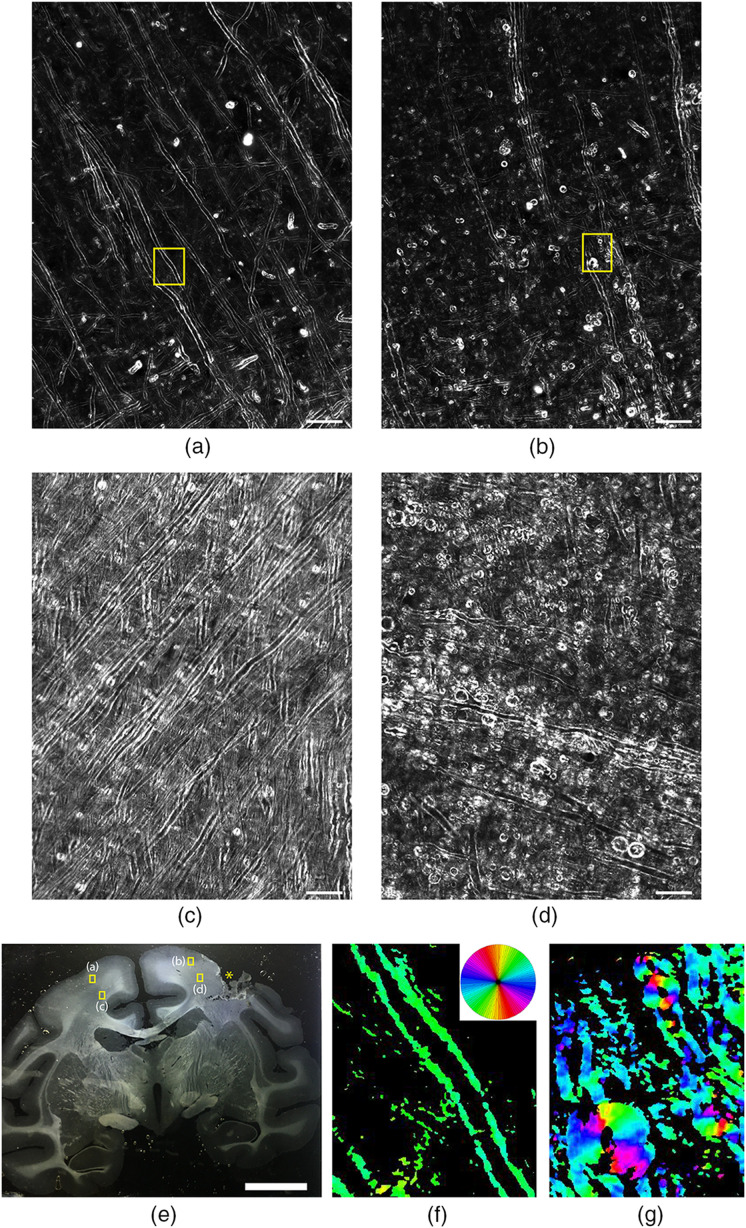
Birefringence microscopy of myelin degeneration following cortical injury in the rhesus monkey. CCP-BRM displayed for images (a)–(d), and qBRM for (f) and (g). (a) Healthy and (b) damaged gray matter regions. (c) Healthy and (d) damaged white matter regions (20× objective, 0.5 NA). Scale bars: 50  μm. (e) Photograph of tissue section, with regions shown in (a)–(d) indicated with yellow boxes and the location of the lesion indicated with a yellow asterisk. Scale bar: 1 cm. (f) Healthy and (g) damaged qBRM images for the regions shown with yellow boxes in (a) and (b) (40× objective, 0.75 NA). In-plane optic-axis orientation indicated by the color scale shown in the upper-right corner of (f).

Myelin status in whole-brain histological sections was analyzed with birefringence microscopy in tissues of a young monkey (6 years old) with injury to a region of primary motor cortex of one hemisphere 6 weeks before euthanization. In this animal, we observed a striking difference in the structure of myelin when comparing the damaged region of the motor cortex and the corresponding region in the contralateral hemisphere. In the hemisphere unaffected by the cortical injury, gray [Fig. 5(a)] and white [Fig. 5(c)] matter regions appear normal, with myelinated axons being completely intact. In perilesional gray [Fig. 5(b)] and white [Fig. 5(d)] matter, myelin exhibits clear signs of structural breakdown, as much of the myelin has formed into vesicular debris structures, indicating it is no longer associated with axons. The CCP-BRM images captured in gray matter [Figs. 5(a), 5(b)] are 2D projections generated through focus-stacking, as described above, whereas the images captured in white matter [Figs. 5(c), 5(d)] are from a single plane since these regions are much more myelin-dense. An image of the brain section, showing the location of the lesion (yellow asterisk) and the regions of interest (yellow boxes) for Figs. 5(a)–5(d), is provided in Fig. 5(e). The images of myelin taken near the lesion show that large amounts of myelin debris remained at the time of death. This myelin debris is widespread in the perilesional space and can be seen to extend for several millimeters from the site of the lesion. In similar areas on the contralateral side and within different anatomical sections, myelin appears healthy and normal.

To demonstrate the quantitative advantages of birefringence microscopy for characterizing myelin debris, qBRM was also performed on this sample field, and the optic-axis orientation maps are shown for the regions of interest (yellow boxes) shown in Figs. 5(a) and 5(b). In normal myelinated axons [Fig. 5(f)], the orientation of the myelin optic axis (given by the color scale shown in the upper-right corner) is consistent along the length of the myelin sheath, whereas in degenerated myelin [Fig. 5(g)], the optic axis reorients variously, as myelin forms into debris structures. Based on these features, we anticipate that qBRM will aid in the development of new quantitative metrics for evaluating the extent of myelin pathology, as well as the clearance of myelin debris and regeneration.

## 4 Discussion

Injury to the brain due to stroke or trauma is a leading cause of disability. In ischemic stroke, the most severely affected area of the brain undergoes necrosis, with the size of the necrotic lesion depending on the size of the vascular network that was disrupted. Following the death of neurons in these regions, entire networks of myelinated axons undergo Wallerian degeneration, leading to the accumulation of vast amounts of myelin debris. When myelin debris cannot be efficiently cleared by microglia, it results in inhibitory effects on regeneration and remyelination, limiting functional recovery.^22^^,^^65^^–^^67^

In animal models of stroke, significant improvements in functional recovery have been demonstrated with a variety of cell-based therapies^68^ and, more recently, with extracellular vesicles (EVs) derived from mesenchymal stem cells (MSCs).^69^^,^^70^ The EVs likely supply damaged tissue with growth factors that promote structural regeneration. In addition, evidence suggests that MSC-derived EVs minimize secondary damage due to inflammation^71^ and may limit further myelin degeneration around the lesion. However, investigation of these mechanisms has been impeded by the inability to assess clearance of myelin debris and remyelination in the perilesional space, due to lack of methods to quantitatively image myelin structure across whole-brain sections.

Here, we report effective imaging of myelin structure in brain sections with two complementary configurations of birefringence microscopy: (1) CCP-BRM and (2) qBRM. CCP-BRM provides a simple method for performing real-time imaging, enabling the user to efficiently survey myelin across large brain sections and to home-in on particular regions of interest. While other groups have approached real-time birefringence microscopy by achieving fast quantitative imaging speeds with liquid-crystal variable retarders,^59^^,^^60^ these strategies are fundamentally limited by the requirement of at least five images plus image postprocessing to generate birefringence images.^51^^,^^72^^,^^73^ With the system we have implemented, continuous image processing is not required to view myelin birefringence, as we have demonstrated that the high-contrast images of myelin taken with CCP-BRM closely resemble and provide similar information content to the retardance images generated from qBRM. As such, CCP-BRM is a powerful tool for rapid histopathological assessment of myelin structure. Most importantly, this technique enables efficient and user-friendly volumetric imaging of myelin, allowing the profiles of individual myelinated axons to be followed through the entire thickness of a tissue section as thick as 50  μm, thus facilitating the differentiation of myelin debris from transverse myelinated axons, which manifest as similar circular cross-sections within a single focal plane.

With the framework we have designed, once regions of interest have been identified and imaged with CCP-BRM, qBRM can be employed for high-resolution quantitative imaging of myelin structure. With qBRM, the retardance and optic-axis orientation are determined for each pixel in the image, with all pixels measured in parallel, providing quantitative information that facilitates structural comparisons between myelinated axons and myelin debris over large fields of view. With minimal sacrifice of imaging speed during qBRM (compared to systems using electronically controlled liquid-crystal elements), we submit that our system, designed around the use of zero-order quarter-wave plates and fast motorized linear polarizers, is superior for high-contrast imaging of myelin with CCP-BRM while also enabling efficient and automated qBRM.

In this study, we demonstrate that birefringence microscopy is selective for myelin and is a valuable technique for histopathological assessment of myelin structural breakdown. We illustrate the benefits of these methods by imaging the myelin breakdown associated with cortical injury in monkeys, as a model for stroke. In a monkey that was allowed to recover for 6 weeks following injury, birefringence microscopy reveals the accumulation of large amounts of vesicular myelin debris in perilesional white and gray matter. By analyzing the presence of myelin debris structures in brain tissue around the lesion, the extent of the injury can be rapidly determined with CCP-BRM and further quantified with qBRM. In future studies of recovery from cortical damage, we propose that birefringence microscopy will be useful in identifying areas susceptible to myelin debris-induced inflammation, and in the analysis of neurorestorative therapies and their capacity to promote structural reorganization and remyelination. In addition to these applications in stroke research, birefringence microscopy will likely provide new insights into myelin degradation seen in demyelinating diseases and neurotrauma and may find similar utility in the study of the more subtle myelin defects that occur in other neurodegenerative conditions, such as aging and AD.
